# Cost-effectiveness of Pembrolizumab as a Second-Line Therapy for Hepatocellular Carcinoma

**DOI:** 10.1001/jamanetworkopen.2020.33761

**Published:** 2021-01-19

**Authors:** Chi-leung Chiang, Sik-kwan Chan, Shing-fung Lee, Irene Oi-ling Wong, Horace Cheuk-wai Choi

**Affiliations:** 1 Department of Clinical Oncology, University of Hong Kong, Hong Kong (SAR), China; 2 Department of Clinical Oncology, Tuen Mun Hospital, Hong Kong (SAR), China; 3 School of Public Health, University of Hong Kong, Hong Kong (SAR), China

## Abstract

**Importance:**

Immune checkpoint inhibitors have been approved for use as a second-line therapy for hepatocellular carcinoma (HCC) in patients who previously received sorafenib. Pembrolizumab has shown substantial antitumor activity and a favorable toxicity profile as a second-line treatment of HCC. However, considering the high cost of pembrolizumab, there is a need to assess its value by considering both the clinical efficacy and cost.

**Objective:**

To evaluate the cost-effectiveness of pembrolizumab vs placebo as second-line therapy in patients with HCC from the US payer perspective.

**Design, Setting, and Participants:**

A Markov model was developed to compare the lifetime cost and efficacy of pembrolizumab as a second-line treatment of HCC with those of placebo using outcome data from the KEYNOTE-240 randomized placebo-controlled trial, which included 413 patients with advanced HCC previously treated with sorafenib and randomized patients to receive pembrolizumab plus best supportive care or placebo plus best supportive care in a 2:1 ratio.

**Main Outcomes and Measures:**

Life-years, quality-adjusted life-years (QALYs), lifetime costs, and incremental cost-effectiveness ratio (ICER) were estimated at a willingness-to-pay threshold of $150 000 per QALY. One-way and probabilistic sensitivity analyses were performed to account for the parameter of uncertainty. A cost-threshold analysis was also performed. The study was conducted from January 31 to July 29, 2020.

**Results:**

The base-case model found that treatment with pembrolizumab was associated with increased overall cost by $47 057 and improved effectiveness by 0.138 QALYs compared with placebo, leading to an ICER of $340 409 per QALY. The model was most sensitive to the hazard ratio of overall survival (range, 0.61-1.00), health utility of placebo (range, 0.59-0.93), price of pembrolizumab (range, $5531-$8297), and price of postprogression therapies (range, $5596-$7944 for pembrolizumab and $4770-$7156 for placebo). The ICER of pembrolizumab was larger than $150 000 per QALY in most of the sensitivity and subgroup analyses. The price of pembrolizumab needed to be reduced by 57.7% to $2925 per cycle to achieve cost-effectiveness.

**Conclusions and Relevance:**

The findings of this cost-effectiveness analysis suggest that, at its current price, pembrolizumab is not a cost-effective second-line therapy for HCC in the US, with a willingness-to-pay threshold of $150 000 per QALY.

## Key Points

**Question:**

Is pembrolizumab a cost-effective second-line therapy for advanced hepatocellular carcinoma from the US perspective?

**Findings:**

In this economic evaluation of data from the KEYNOTE-240 randomized trial, the incremental number of quality-adjusted life-years gained in the base-case scenario using pembrolizumab was 0.138 years at a cost of $340 409 per quality-adjusted life-year compared with that gained using placebo, which exceeded the willingness-to-pay threshold of $150 000 per quality-adjusted life-year.

**Meaning:**

This economic evaluation model suggests that pembrolizumab is not a cost-effective second-line therapy for hepatocellular carcinoma.

## Teaser

This economic evaluation analyzes the cost-effectiveness of pembrolizumab as a second-line treatment in patients with advanced hepatocellular carcinoma.

## Introduction

Hepatocellular carcinoma (HCC) is the most common primary liver cancer and a leading cause of cancer-related death globally.^1^ Most patients present with advanced disease, for which highly effective treatments are lacking. Sorafenib, an antiangiogenic multikinase inhibitor, has been the only approved systemic treatment for HCC for more than a decade.^2,3^ Several new antiangiogenic agents have been approved for the treatment of advanced HCC. These agents include lenvatinib as a first-line therapy and regorafenib, cabozantinib, and ramucirumab as second-line treatments after sorafenib therapy.^4,5,6,7^ However, the survival benefit and tumor response of these second-line treatments were usually limited.^4,5,6,7^ Previous studies suggested that tyrosine kinase inhibitors were not cost-effective second-line treatments in patients with advanced HCC.^8,9^

Pembrolizumab and nivolumab are monoclonal antibodies that bind to anti–programmed death 1 (PD-1) receptor, thus restoring the T-cell immune activity directed against tumor cells. Anti–PD-1 therapy has shown substantial clinical efficacy and a promising safety profile in patients with HCC who have been treated with sorafenib in 2 single-arm trials, leading to the accelerated approval of both drugs by the US Food and Drug Administration (FDA).^10,11^

The KEYNOTE-240 study was the first randomized phase 3 trial of anti–PD-1 therapy in patients with HCC. In this trial, patients previously treated with sorafenib were randomized to the pembrolizumab or best supportive care groups.^12^ Although the results of this study did not meet the prespecified statistical criteria for the co-primary end points of overall survival and progression-free survival, the magnitude of the survival benefit (13.9 vs 10.6 months; hazard ratio, 0.78; *P* = .02) and the response rate (18.3% vs 4.4%; *P* = .00007) were favorable for pembrolizumab.^12^ In addition, immune checkpoint inhibitors generally exhibit a good toxicity profile compared with that of tyrosine kinase inhibitors.^1^ Overall, the current data support that pembrolizumab is a favorable second-line treatment option for patients with HCC. In this study, we aimed to evaluate the cost-effectiveness of pembrolizumab vs placebo as a second-line therapy for patients with advanced HCC from the US payer perspective.

## Methods

### Model Overview

This economic evaluation model-based study used published trial data with no identifiable patient data involved and was exempted from institutional review board approval by The University of Hong Kong/Hospital Authority Hong Kong West Cluster. Our study followed the Consolidated Health Economic Evaluation Reporting Standards (CHEERS) reporting guideline for economic evaluations.^13^ The present study was conducted from January 31 to July 29, 2020.

**Figure 1.**
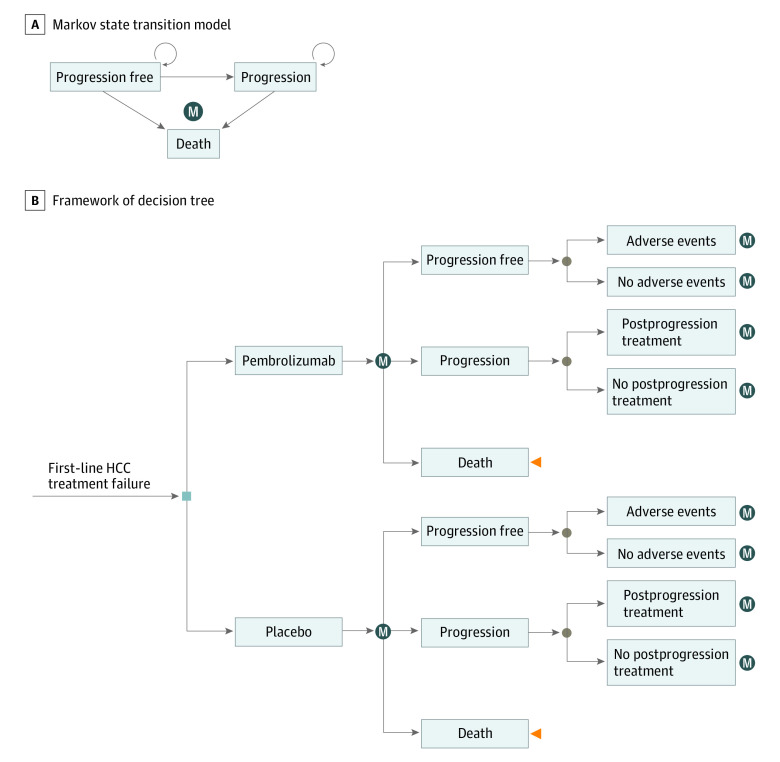
Model Structure A, Markov state transition model. B, Framework of decision tree. HCC indicates hepatocellular carcinoma; M, Markov node.

In the KEYNOTE-240 trial, eligible patients had Barcelona Clinic Liver Cancer stage C or stage B disease that was not amenable or refractory to locoregional therapy. Patients had Child-Pugh class A liver function. The 413 participants were randomly assigned to receive pembrolizumab plus best supportive care vs placebo plus best supportive care in a 2:1 ratio. We developed a 3-state Markov model (progression-free disease, postprogression disease, and death) to estimate the costs and the cost-effectiveness of the second-line treatments for HCC (Figure 1). Two treatment options were evaluated: pembrolizumab, 200 mg, vs placebo administered intravenously in an every-3-week cycle. We assumed that the treatments were continued until disease progression, until unacceptable toxic effects, or up to 35 cycles (2 years), whichever was earliest. Upon progression of the disease, both groups could receive subsequent treatment until death. All patients in each health state could experience progression to death; all patients who died were assumed to receive end-of-life care.

The model included only the direct medical care costs. Each 3-weekly model cycle was consistent with that in the KEYNOTE-240 trial. Half-cycle correction was applied to reduce the bias in our discrete-time model. In addition, we adopted 3% annual discount rates for both the costs and health outcomes. The time horizon was 3 years considering the limited life expectancy of patients with HCC receiving second-line therapy.

The primary outcomes included the total cost, life-year, quality-adjusted life-year (QALY), and incremental cost-effectiveness ratio (ICER), which was defined as the incremental costs divided by the incremental health outcomes. We considered a willingness-to-pay (WTP) threshold, expressed in US dollars, at $150 000 per QALY gained for considering pembrolizumab to be cost-effective.

### Survival and Progression Risk Estimates

The overall probability of death in any state included the probability of death from HCC and the background mortality rate from other causes. The probability of death from HCC (ie, disease progression to death) for each treatment strategy was estimated based on the overall survival curves of the KEYNOTE-240 trial.^11^ We first reconstructed the published overall survival curves in the KEYNOTE-240 trial by generating the pseudoindividual patient data using the algorithm developed by Hoyle and Henley^14^ and Wan et al^15^ (eFigure 1 in the Supplement). We then fit the reconstructed overall survival curves to Weibull distribution, which showed the best fit with the smallest Akaike information criterion compared with other distributions, such as exponential, log-logistic, and log-normal distributions; eTable 1 in the Supplement provides the Akaike information criteria of the tested distributions. We estimated the disease progression risk using the same approach by fitting the reconstructed progression-free survival curves from the KEYNOTE-240 trial.^12^ The background mortality rate (ie, from progression-free disease to death) for each age group was based on US life tables (eTable 2 in the Supplement).^16^

The KEYNOTE-240 study reported survival at 30 months through initiation of treatment; however, the survival of responders to immunotherapy beyond 30 months remains unknown owing to limited long-term follow-up data. Therefore, we assessed the uncertainties using scenario analysis. In base-case analysis, we estimated survival beyond 30 months based on extrapolations of survival curves in the KEYNOTE-240 study. Previous studies have suggested that immunotherapy may cure a proportion of patients and extrapolation of long-term outcomes based on a standard parametric model may lead to inaccurate assessments^17,18,19^; therefore, a more optimistic scenario was assumed that patients alive after 30 months were cured of HCC, and their risk of death returned to their normal age-adjusted mortality risk.^16^ We estimated that 25.0% of the patients were alive at 30 months.^12^

### Utility Estimates

Because the KEYNOTE-240 study did not report quality-of-life data, we used quality-of-life data from a recently published study of pembrolizumab in bladder cancer.^20^ The health utilities of placebo-treated patients were obtained from the placebo arm of the RESORCE trial, which evaluated the use of regorafenib in a clinical scenario similar to that of the KEYNOTE-240 trial.^8^ Quality-of-life utilities were assigned to patients who received pembrolizumab (0.84) and placebo (0.76). We specified a utility weight of 0.68 for patients with disease progression, which was derived from the National Institute for Health and Clinical Excellence technology appraisal guidance.^21^ The uncertainty surrounding utility values was evaluated in a sensitivity analysis. We also considered utility decrements for each episode of developing grade 3 or 4 toxic effects.^22,23,24^

### Cost Estimates

Direct medical costs included the costs of drugs, drug administration, and management of adverse events. We considered grade 3 to 4 adverse effects that had significantly different rates between treatments in the KEYNOTE-240 trial and included fatigue, nausea, hypothyroidism, pneumonitis, skin reactions, hepatitis, colitis, diabetes, and hypophysitis.^12^

According to the KEYNOTE-240 trial, posttreatment anticancer medications were administered to 41.7% of patients who received pembrolizumab and 47.4% of those who received placebo; among them, 6.8% of patients in the pembrolizumab group and 10.4% of those in the placebo group received anti–PD-1/anti–programmed cell death ligand 1 (PD-L1) therapy. We then modeled patients as receiving different types of targeted therapies and unapproved medications similar to those detailed in previous randomized trials.^6,7^ The duration of each postprogression therapy was obtained from published trials.^5,6,7,10^ All patients who died in both arms of the trial were assumed to have received equivalent end-of-life care.^25^

We used the 2020 average sale price from the Centers for Medicare & Medicaid Services to estimate the unit price of drugs.^26,27^ Following the approach used by Goldstein et al,^28^ administration-related costs were estimated according to the Medicare physician fee schedule for 2020. Costs from adverse effects were estimated based on published data from patients receiving treatment of various neoplasms.^29,30,31^ We adjusted all of the above costs for inflation to reflect the 2020 US dollar value using the US consumer price index.^26^

### Sensitivity Analysis and Threshold Analysis

In a 1-way sensitivity analysis, we obtained the lower and upper limits from the CIs of the corresponding estimates or ±20% of the base-case values and tested them over plausible ranges (eTable 3 in the Supplement). In 2-way sensitivity analyses, we tested the interplay between the median survival times, utility values, and postprogression therapy costs for the pembrolizumab and placebo arms. We further conducted probabilistic sensitivity analyses by 10 000 Monte Carlo simulations, with the variables simultaneously varied with prespecified distributions (eTable 3 in the Supplement).^32^ We also performed the threshold analysis and subgroup analysis based on the subgroup findings presented in the forest plot of the KEYNOTE-240 trial.^12^ Furthermore, we conducted scenario analyses to assess model sensitivity to changes in long-term survival outcome, the use of a recently approved alternative schedule of pembrolizumab (400 mg every 6 weeks), and longer time horizons (5 years, 10 years, and lifetime).^33,34^

The published survival curves were digitalized (GetDataGraphDigitizer, version 2.25) and restructured (R, version 3.5.1; R Foundation). The cost-effectiveness analysis model was implemented in TreeAge Pro 2020 (TreeAge Software).

## Results

The model estimated that the life expectancy of patients receiving pembrolizumab was 1.324 life-years, which was 0.153 life-years longer than that of patients receiving placebo (Table 1). After accounting for the quality-of-life adjustment, patients receiving pembrolizumab had 0.954 QALYs, which was 0.138 QALYs more than patients receiving placebo. The use of pembrolizumab cost an additional $47 057, resulting in an ICER of $308 377 per life-year, or $340 409 per QALY, compared with that of patients receiving placebo.

**Table 1.** Summary of Base-Case Results

Parameter	Treatment	
	Placebo	Pembrolizumab
Total cost, $	207 589	254 646
Incremental total cost		47 057
QALYs	0.816	0.954
QALY gain		0.138
Life-years	1.171	1.324
Incremental life-year		0.153
ICER, $ (pembrolizumab vs placebo)		
Per life-year	NA	308 377
Per QALY	NA	340 409

Abbreviations: ICER, incremental cost-effectiveness ratio; NA, not applicable; QALY, quality-adjusted life-year.

**Figure 2.**
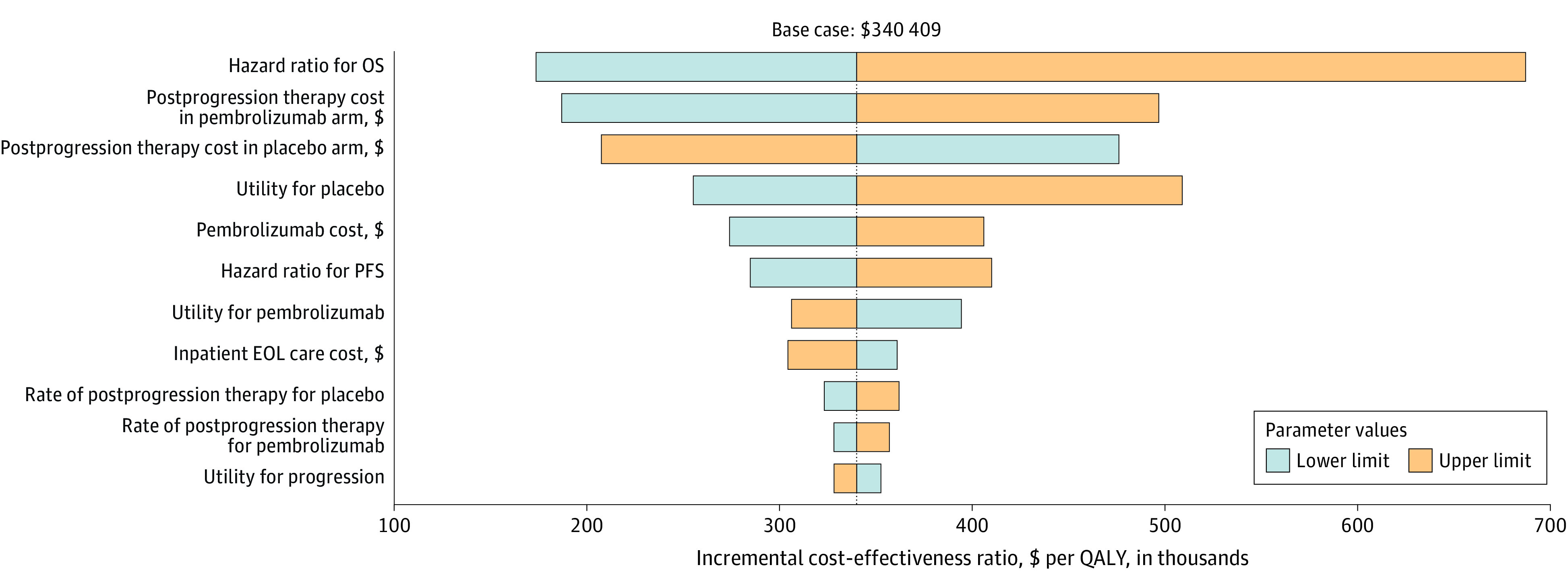
One-Way Sensitivity Analysis for Using Pembrolizumab vs Placebo EOL indicates end-of-life; OS, overall survival; PFS, progression-free survival; and QALY, quality-adjusted life-year.

Figure 2 shows the results of the 1-way sensitivity analysis. The variables with the greatest influence on the ICER were the hazard ratios for overall survival (0.78; range, 0.61-1.00), the utility of placebo (0.76; range, 0.59-0.93), the price of postprogression therapy in the pembrolizumab arm ($6620 per cycle; range, $5596-$7944), the price of postprogression therapy in the placebo arm ($5963 per cycle; range, $4770-$7156), and the price of pembrolizumab ($6915 per cycle; range, $5531-$8297). Along the broad variations in the range for each parameter, the ICERs remained above $150 000 per QALY gained.

**Figure 3.**
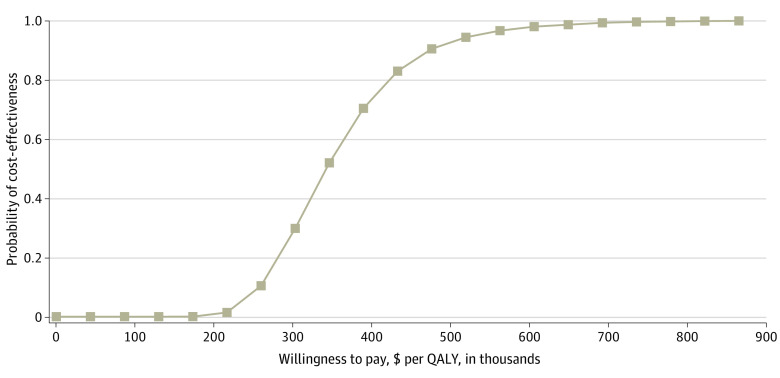
Cost-effectiveness Acceptability Curve of Using Pembrolizumab QALY indicates quality-adjusted life-year.

Regarding probabilistic sensitivity analyses, the cost-effectiveness acceptability curve (Figure 3) showed 0% acceptability of using pembrolizumab at a WTP value of $150 000 per QALY. If we set a much higher WTP level of more than $350 000 per QALY, the probability of the use of pembrolizumab being cost-effective would be above 50%. The results of 2-way sensitivity analyses demonstrated that the ICERs were above $150 000 per QALY across most median survival, utility, and postprogression therapy cost combinations; however, the ICERs were below $150 000 per QALY when the median overall survival of pembrolizumab was more than 12 months longer than that of placebo or when the postprogression therapy cost of pembrolizumab was more than $1200 per cycle less than that of placebo (eFigure 2 in the Supplement).

In scenario analyses (Table 2), if we assume a more optimistic outcome in which patients alive at the end of the study no longer had HCC, the ICER of pembrolizumab decreased to $222 109 per QALY. Similarly, although increasing the time horizon of the model or using pembrolizumab, 400 mg, every 6 weeks also improved the economic value of pembrolizumab, the ICER remained above $150 000 per QALY.

**Table 2.** Scenario Analyses

Parameter	ICER ($/QALY)
Base-case scenario	
Long-term OS extrapolated from survival curves^35^	340 409
Pembrolizumab schedule: 200 mg every 3 wk	
Time horizon: 3 y	
Scenario analysis	
Long-term OS estimation	
Optimistic scenario: Patients alive after 30 mo were cured of HCC	222 109
Pembrolizumab schedule	
Alternative schedule: 400 mg every 6 wk	286 884
Time horizon	
5 y	233 064
10 y	197 305
Lifetime	196 317

Abbreviations: HCC, hepatocellular carcinoma; ICER, incremental cost-effectiveness ratio; OS, overall survival; QALY, quality-adjusted life-year.

Subgroup analyses (eTable 4 in the Supplement) showed that pembrolizumab as a second-line therapy was not cost-effective in all the subgroups at WTP values of $150 000 per QALY. For the same threshold, only patients with macrovascular invasion had a 5.7% chance that pembrolizumab would be a cost-effective option.

### Threshold Analysis

In the threshold analysis for use of pembrolizumab to be cost-effective compared with placebo based on the WTP level of $150 000 per QALY, the listed price of pembrolizumab would have to be reduced by 57.7% to $2925 per cycle from its original price of $6915 per cycle.

## Discussion

To our knowledge, we performed the first study to examine the cost-effectiveness of immunotherapy in patients with advanced HCC. Based on our model, pembrolizumab provided modest incremental benefits at high incremental costs per QALY gained. The probabilistic sensitivity analysis suggests that pembrolizumab was unlikely to be cost-effective at the commonly adopted WTP threshold of $150 000 per QALY gained, as recommended by Neumann et al.^36^

Pembrolizumab can provide 0.138 additional QALYs compared with QALYs provided by placebo; however, pembrolizumab is not a cost-effective second-line treatment. The most influential factors of our model were survival benefit, price of pembrolizumab, price of postprogression therapies, and the utility of placebo. Even the KEYNOTE-240 study reported the larger survival advantages of pembrolizumab in 2 post hoc analyses that adjusted for a higher rate of postprogression therapy in the placebo arm (survival benefit of 3-4.6 months)^12^; however, the 2-way sensitivity analysis suggested that a survival benefit longer than 12 months is needed for pembrolizumab to be cost-effective. In addition, even if we assumed an optimistic scenario that pembrolizumab could cure patients, the treatment failed to cross the threshold of cost-effectiveness. With the price at the time of the study, subgroup analyses demonstrated that anti–PD-1 therapy was not cost-effective in all patient subsets; pembrolizumab needs a substantial reduction of price (57.7% reduction) for it to become cost-effective.

We lacked robust head-to-head trial data to compare various second-line therapies.^5,6,10^ However, regorafenib has been shown to have an ICER of $224 362 per QALY; the value of cabozantinib was even over $1 million per QALY gained.^8,9^ To our knowledge, there are no published data on the value of ramucirumab, yet it is unlikely to be a cost-effective agent given its modest survival advantage (8.5 months for ramucirumab vs 7.3 months for placebo) even in a selected population of patients receiving α-fetoprotein, 400 ng/mL or more, and its expensive cost.^7^ Little is known about the value of nivolumab, another anti–PD-1 agent; however, given that 2 checkpoint inhibitors have similar efficacy (median overall survival, 15.6 months [nivolumab] vs 13.9 months [pembrolizumab]) and wholesale price (6-week cost of $20 020 for nivolumab, 240 mg, every 2 weeks vs $19 755 for pembrolizumab, 200 mg, every 3 weeks),^26,27^ our sensitivity analyses suggest that anti–PD-1 therapy is unlikely to be cost-effective in such a range of survival benefit and price. Overall, it appears that most of the approved second-line therapies for HCC provide low economic value at their current costs. The FDA has recently granted an accelerated approval of the combination of nivolumab and ipilimumab as second-line therapy for HCC based on its promising results in the phase 1/2 CheckMate 040 study.^37^ Further randomized trials and cost-effectiveness analysis of this regimen are warranted.

In light of the low cost-effectiveness of the currently approved second-line therapies of HCC, apart from developing a better regimen, efforts should also focus on identification of predictive biomarkers. However, unlike in other cancers, PD-L1 expression was not predictive of response to pembrolizumab in patients with HCC.^11^ Further research is therefore needed to discover the biomarkers to aid in patient selection that would in turn improve the value of anti–PD-1 therapy. A recent study suggested that transforming growth factor-β could be a potential biomarker.^38^ Among 29 patients with advanced HCC receiving pembrolizumab, the median overall survival of those receiving transforming growth factor-β, 200 pg/mL or more, was significantly better than in those receiving transforming growth factor-β, less than 200 pg/mL (7 months vs 25 months, *P* = .005). Validation of the findings in a larger sample size is warranted.

With increasing drug prices, it has become a paradigm that the new oncology products failed to cross the commonly used WTP threshold for cost-effectiveness. Because the FDA does not consider cost-effectiveness in its decision of approval, the cost-effectiveness analyses often end up as an academic exercise. For the situation to be changed, the oncology community should consider the cost-effectiveness of treatments in their guideline recommendations. For example, the FDA-approved agent necitumumab was deleted from the National Comprehensive Cancer Network guidelines (non–small cell lung cancer) based on its poor cost-effectiveness.^39^ Also, the pricing and payment systems should be reformed. The response to new immuno-oncology products varies widely. Some payers and patient advocates have proposed performance-based pricing, which allows all stakeholders to share the financial risk of therapy of uncertain long-term outcomes, and therefore provides economic incentives for the payers to cover the expensive therapy.^40^

### Limitations

Several limitations exist in our study. First, any biases within the KEYNOTE-240 trial that affect its validity and generalizability will impact our study. Previous studies have highlighted the difference in patients with HCC enrolled in clinical trials vs those in real-world settings.^41^ Similarly, patients enrolled in KEYNOTE-240 were highly selected with excellent performance status.^11^ Thus, the real-world effectiveness of pembrolizumab is likely to be lower, but it would not alter our study conclusion.

Second, our analysis was limited by data availability and assumption. Quality-of-life data on patients receiving pembrolizumab in the KEYNOTE-240 trial were not published; therefore, we extrapolated these data from previously published studies of pembrolizumab.^20^ This process was appropriate because the utility score was similar to that reported in patients with HCC who received nivolumab or regorafenib in the second-line treatment setting.^5,10^ We also assumed the decline of quality of life on progression of HCC, which is consistent with previous literature and real-life experience.^21,41^ We considered the cost of postprogression therapy; however, only the percentage of patients receiving active treatment and anti–PD-1/anti–PD-L1 therapy was reported in the KEYNOTE-240 trial.^12^ We estimated the cost based on the treatment pattern and treatment duration from previous studies of second-line therapy for HCC^5,6,7,10^; our estimated cost was comparable to that in a similar cost-effectiveness study.^9^ We addressed these uncertainties by conducting a series of sensitivity analyses suggesting that the ICER was consistently greater than $150 000 per QALY in most of the utility and postprogression therapy combinations.

Third, our model used extrapolated overall survival and progression-free survival curves based on the KEYNOTE-240 trial. There are inevitably uncertainties, particularly when projecting beyond the trial observation period. One of the strengths of our report is that we have performed a scenario analysis to address this limitation; we demonstrated that pembrolizumab is not cost-effective even under the most optimistic assumption. In addition, the average sales price in the Medicare program was used to estimate the cost of pembrolizumab in our model. We have accounted for the variation of reimbursement rates in a range of drug costs in the sensitivity analyses.

## Conclusions

In this study, from the perspective of US payers, use of pembrolizumab as the second-line therapy with previously received sorafenib was estimated to be not a cost-effective treatment in patients with HCC at the commonly adopted WTP threshold of $150 000 per QALY. The price of pembrolizumab needs to be substantially reduced to become cost-effective.

## Notes

Supplement. **eTable 1.** The Akaike Information Criterion (AIC) Measures of Goodness of Fit for the Comparison of Survival Models **eFigure 1.** Curve Fitting to Reconstructed Survival Curves **eTable 2.** Background Mortality Rate **eTable 3.** Model Parameters, Baseline Values, Ranges, and Distribution for Sensitivity Analyses **eFigure 2. **Two-Way Sensitivity Analysis **eTable 4. **Results of Subgroup Analyses
