# Supplementary material for: Cost-effectiveness of Pembrolizumab as a Second-Line Therapy for Hepatocellular Carcinoma
Source: JAMA Netw Open. 2021 Jan 19;4(1):e2033761. doi: 10.1001/jamanetworkopen.2020.33761 (PMC7816108; doi:10.1001/jamanetworkopen.2020.33761)
Supplement: Supplement. — eTable 1. The Akaike Information Criterion (AIC) Measures of Goodness of Fit for the Comparison of Survival Models eFigure 1. Curve Fitting to Reconstructed Survival Curves eTable 2. Background Mortality Rate eTable 3. Model Parameters, Baseline Values, Ranges, and Distribution for Sensitivity Analyses eFigure 2. Two-Way Sensitivity Analysis eTable 4. Results of Subgroup Analyses [file jamanetwopen-e2033761-s001.pdf]

Supplementary Online Content

Chiang CL, Chan SK, Lee SF, Wong IOL, Choi HCW. Cost-effectiveness of pembrolizumab as a second-line therapy for hepatocellular carcinoma. *JAMA Netw Open*. 2021;4(1):e2033761. doi:10.1001/jamanetworkopen.2020.33761

eTable 1. The Akaike Information Criterion (AIC) Measures of Goodness of Fit for the Comparison of Survival Models

eFigure 1. Curve Fitting to Reconstructed Survival Curves

eTable 2. Background Mortality Rate

eTable 3. Model Parameters, Baseline Values, Ranges, and Distribution for Sensitivity Analyses

eFigure 2. Two-Way Sensitivity Analysis

eTable 4. Results of Subgroup Analyses

© 2021 Chiang C-l et al. *JAMA Network Open.*

eTable 1. The Akaike information criterion (AIC) measures of goodness of fit for the comparison of survival models

	**Pembrolizumab**	**Placebo**
**OS**		
Exponential	1225	638
Weibull	***1192***	***619***
Log-normal	1202	624
Log-logistic	1199	622
**PFS**		
Exponential	517	518
Weibull	***472***	***478***
Log-normal	503	502
Log-logistic	480	480

*A lower AIC value indicates a better fit. Bold-italic cells represent the lowest AIC values for each survival curve and for each group.

© 2021 Chiang C-l et al. *JAMA Network Open.*

eFigure 1. Curve fitting to reconstructed survival curves

A. Weibull fit (red curve) of the observed overall survival curve (blue line) in pembrolizumab arm.

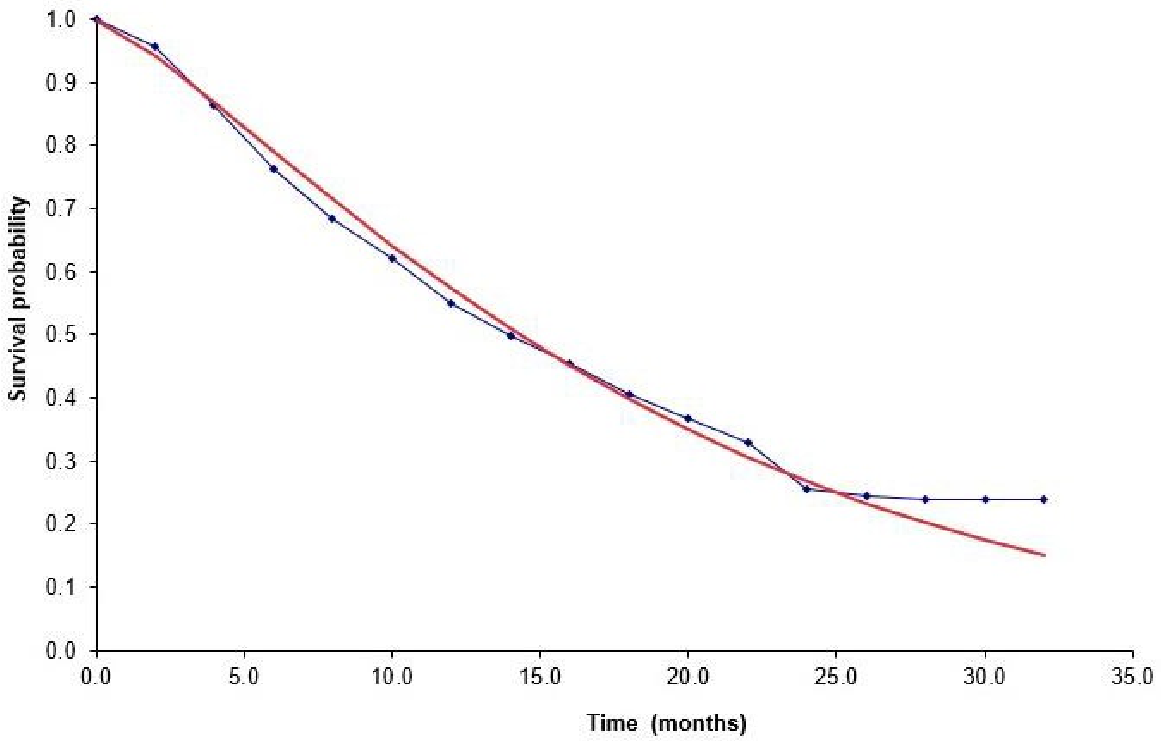

B. Weibull fit (red curve) of the observed overall survival curve (blue line) in placebo arm.

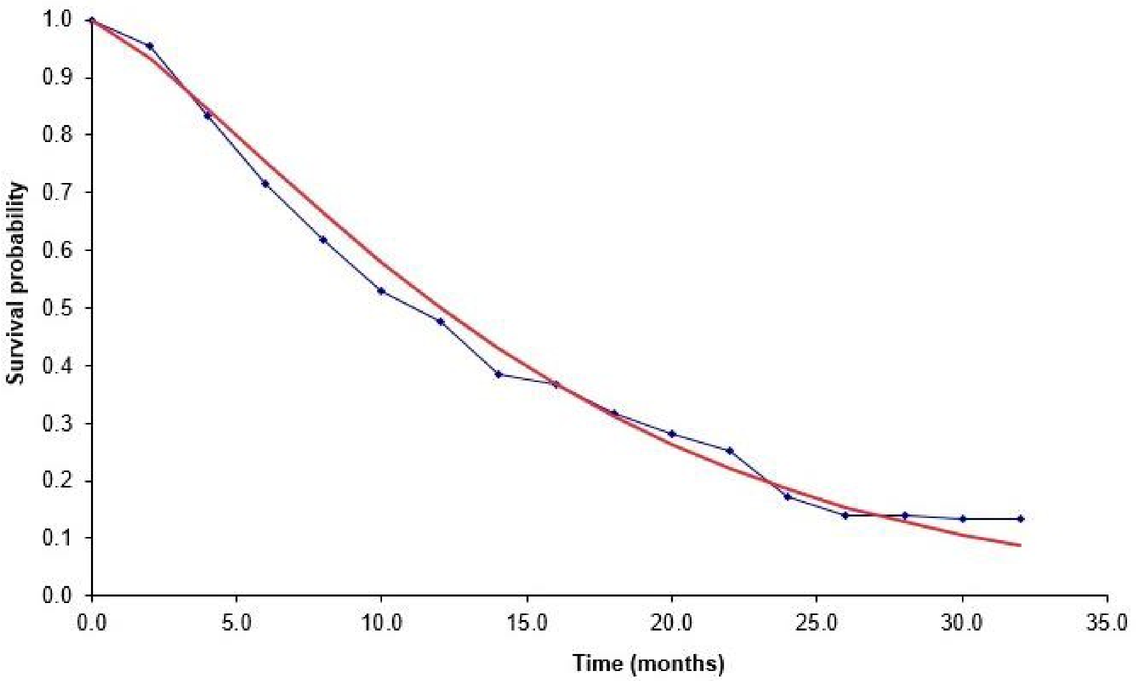

© 2021 Chiang C-l et al. *JAMA Network Open.*

C. Weibull fit (red curve) of the observed progress-free survival curve (blue line) in pembrolizumab arm.

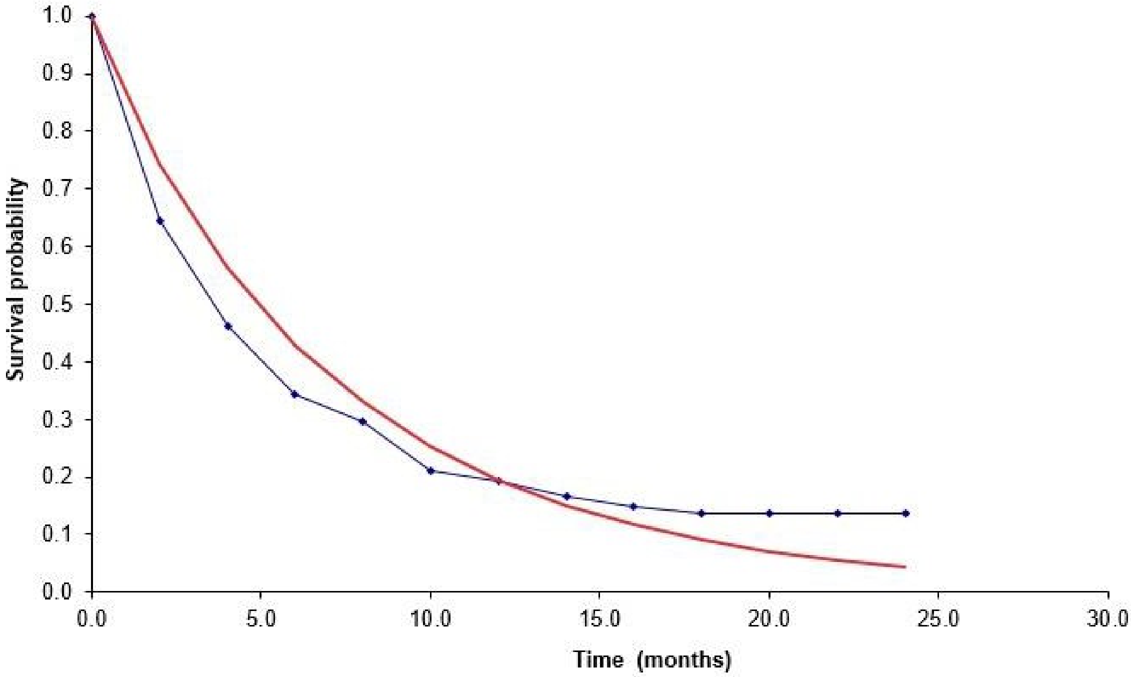

D. Weibull fit (red curve) of the observed progress-free survival curve (blue line) in placebo arm.

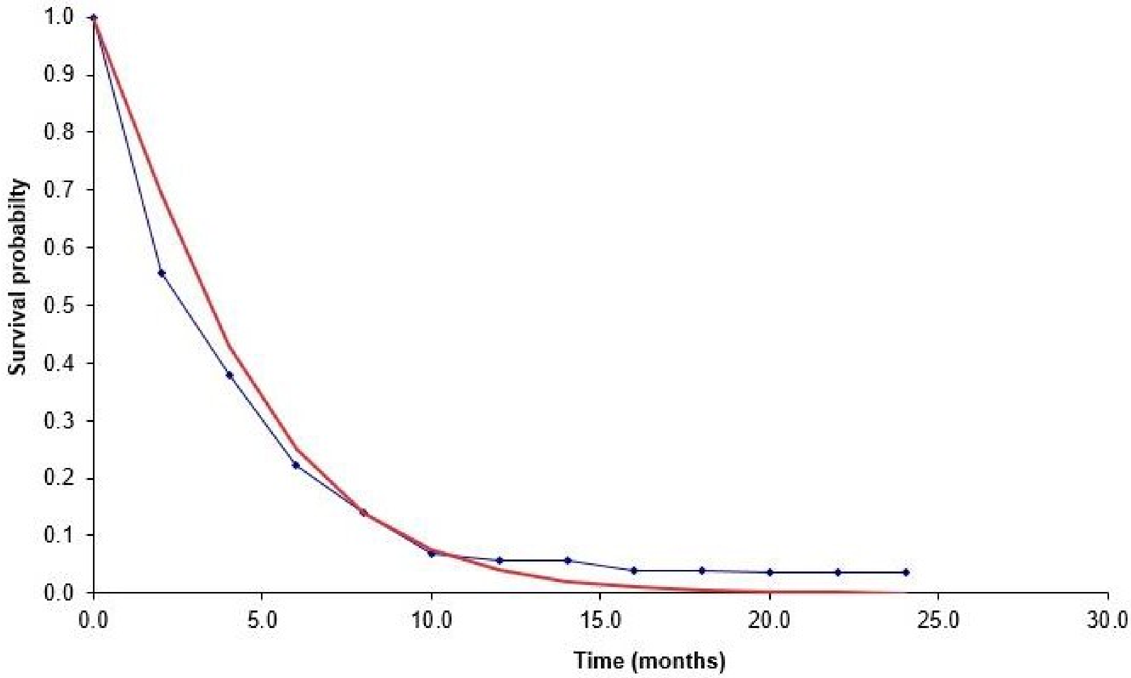

© 2021 Chiang C-l et al. *JAMA Network Open.*

eTable 2. Background mortality rate

Estimates of background mortality rate for each age are provided in the US life table; Arias E, Heron M, Xu J. United States Life Tables, 2019. Natl Vital Stat Rep. 2019; 68:1-65.

Age (years)	Background mortality rate	Age (years)	Background mortality rate	Age (years)	Background mortality rate
18	0.000603	57	0.008857	96	0.291442
19	0.000698	58	0.009542	97	0.314700
20	0.000795	59	0.010285	98	0.338142
21	0.000889	60	0.011098	99	0.361537
22	0.000970	61	0.011952	100	1
23	0.001424	62	0.012814		
24	0.001497	63	0.013657		
25	0.001561	64	0.014502		
26	0.001624	65	0.015384		
27	0.001682	66	0.016444		
28	0.001737	67	0.017624		
29	0.001792	68	0.018968		
30	0.001847	69	0.029586		
31	0.001900	70	0.022109		
32	0.001952	71	0.024359		
33	0.002003	72	0.026347		
34	0.002053	73	0.028810		
35	0.002111	74	0.031309		
36	0.002174	75	0.034486		
37	0.002233	76	0.038026		
38	0.002285	77	0.042286		
39	0.002340	78	0.046547		
40	0.002413	79	0.051534		
41	0.002516	80	0.057008		
42	0.002649	81	0.062923		
43	0.002811	82	0.069911		
44	0.002999	83	0.078099		
45	0.003203	84	0.086754		
46	0.003433	85	0.096549		
47	0.003709	86	0.106472		
48	0.004047	87	0.119677		
49	0.004445	88	0.134128		
50	0.004874	89	0.149846		
51	0.005331	90	0.166829		
52	0.005844	91	0.185047		
53	0.006408	92	0.204441		
54	0.007003	93	0.224919		
55	0.007607	94	0.246354		
56	0.008219	95	0.26890		

© 2021 Chiang C-l et al. *JAMA Network Open.*

eTable 3. Model parameters, baseline values, ranges, and distribution for sensitivity analyses

		One-way sensitivity analysis		Probabilistic sensitivity analysis	
**Parameters**	**Base-case value**	**Lower limit**	**Upper limit**	**Distribution** [a]	**Reference** [b]
**Clinical effectiveness**					
HR for OS (pembro *vs.* placebo)	0.781	0.611	0.998	*lognormal* (0.781, 0.125)	12
HR for PFS (pembro *vs.* placebo)	0.718	0.570	0.904	*lognormal* (0.718, 0.118)	12
Weibull OS model with placebo	$\lambda = 0.028$,<br>$\gamma = 1.287$	-	-	-	
Weibull PFS model with placebo	$\lambda = 0.156$<br>$\gamma = 1.220$	-	-	-	
Rate of post-progression therapy (pembro)	0.417	0.396	0.438	*beta* (882.4, 1233.8)	12
Rate of post-progression therapy (placebo)	0.474	0.45	0.498	*beta* (787.5, 873.9)	12
**Proportion of patients with grade 3-4 AEs**					
**Pembrolizumab**					
Fatigue	0.025	0.02	0.03	*beta* (93.8, 3660.3)	12
Nausea	0.007	0.006	0.008	*beta* (186.8, 26514.3)	12
Hypothyroidism	0.004	0.003	0.005	*beta* (1.7, 411.1)	12
Pneumonitis	0.014	0.011	0.017	*beta* (82.7, 5829.4)	12
Skin reaction	0.022	0.018	0.026	*beta* (113.7, 5059.7)	12
Hepatitis	0.014	0.011	0.017	*beta* (82.7, 5829.4)	12
Colitis	0.007	0.006	0.008	*beta* (186.8, 26514.3)	12
Hypophysitis	0.004	0.003	0.005	*beta* (1.7, 411.1)	12
Type 1 DM	0.004	0.003	0.005	*beta* (1.7, 411.1)	12
**Placebo**					
Fatigue	0.015	0.012	0.018	*beta* (94.8, 6227.7)	12
Nausea	0.007	0.006	0.008	*beta* (186.8, 26514.3)	12
Hypothyroidism	0	0	0	Fixed at 0	12
Pneumonitis	0	0	0	Fixed at 0	12
Skin reaction	0	0	0	Fixed at 0	12
Hepatitis	0	0	0	Fixed at 0	12
Colitis	0	0	0	Fixed at 0	12
Hypophysitis	0	0	0	Fixed at 0	12
Type 1 DM	0.007	0.006	0.008	*beta* (186.8, 26514.3)	12

© 2021 Chiang, C-l, et al. *JAMA Network Open.*

Rate of treatment discontinuation (pembro)	0.172	0.138	0.206	*beta* (81.2, 391.3)	12
Rate of treatment discontinuation (placebo)	0.09	0.07	0.11	*beta* (70.9, 716.9)	12
**Cost parameters**					
Pembrolizumab 200 mg (every 3 weeks)	6,914.46	5531.57	8297.35	*gamma* (96.3, 71.8)	27-28
Drug administration (every 3 weeks)	433.79	347.03	520.55	*gamma* (96.2, 4.5)	27-28
CT imaging (every 6 weeks)	1539	1231	1847	*gamma* (96.1, 16.0)	27
Other care (every week)	174	139.2	208.8	*gamma* (96.3, 1.8)	8
In-patient EOL care	7339	6191	9287	*gamma* (136.4, 53.8)	9
Management of Grade 3-4 AEs					
Fatigue	0	0	0	Fixed at 0	30-32
Nausea	389.03	344.29	433.77	*gamma* (290.2, 1.3)	30-32
Hypothyroidism	21.32	18.76	23.86	*gamma* (268.7, 0.1)	30-32
Pneumonitis	826.90	727.66	926.1	*gamma* (266.6, 3.1)	30-32
Skin reaction	376.39	361.38	474.19	*gamma* (838.1, 0.5)	30-32
Hepatitis	549.75	486.47	612.91	*gamma* (290.3, 1.9)	30-32
Colitis	1003.99	888.51	1119.42	*gamma* (290.2, 3.5)	30-32
Hypophysitis	1614.38	1428.71	1800.02	*gamma* (290.2, 5.6)	30-32
Type 1 DM	1054.24	927.72	1180.72	*gamma* (266.6, 4.0)	30-32
Post-progression therapy cost (every 3 weeks) (pembro)	6620	5296	7944	*gamma* (96.3, 68.7)	30-32
Post-progression therapy cost (every 3 weeks) (placebo)	5963	4770	7156	*gamma* (96.2, 62.0)	30-32
**Utilities and dis-utilities**					
Pembrolizumab without AEs	0.84	0.79	0.88	*beta* (217.4, 41.8)	11
Placebo without AEs	0.76	0.59	0.93	*beta* (19.0, 6.0)	9
HCC, progression	0.68	0.54	0.82	*beta* (28.2, 13.2)	22, 24, 26
Fatigue	0.11	0.08	0.14	*beta* (46.1, 373.0)	23-25
Nausea	0.13	0.09	0.15	*beta* (149.8, 1002.7)	23-25
Hypothyroidism	0	0	0	Fixed at 0	23-25
Pneumonitis	0.17	0.11	0.22	*beta* (36.5, 178.6)	23-25
Skin reaction	0.13	0.09	0.15	*beta* (149.8, 1002.7)	23-25
Hepatitis	0.17	0.11	0.22	*beta* (36.5, 178.6)	23-25
Colitis	0.17	0.12	0.22	*beta* (36.8, 180.0)	23-25
Hypophysitis	0.17	0.11	0.22	*beta* (36.5, 178.6)	23-25
Type 1 DM	0.17	0.11	0.22	*beta* (36.5, 178.6)	23-25

Abbreviations: AE, adverse event; CT, computed tomography; DM, Diabetics mellitus; EOL, end-of-life; HR, hazard ratio; Pembro, pembrolizumab; PFS,

© 2021 Chiang, C-l, et al. *JAMA Network Open.*

progression-free survival; OS, overall survival

[a] Gamma distribution *gamma* (shape, scale) assumed for costs, beta distribution *beta* (α, β) assumed for proportion of AEs and health utilities, and *lognormal* (μ, σ^2) assumed for HRs with μ as the base-case HR and σ as the standard deviation of the natural logarithm.

[b] The reference numberings refer to references in the main text.

© 2021 Chiang, C-l, et al. *JAMA Network Open.*

eFigure 2. Two-way sensitivity analysis

The below shows the change in incremental cost-effectiveness ratios (ICERs) for the two-way sensitivity analysis by (A) median overall survival, (B) utility value at progression-free state, and (C) post-progression therapy cost between pembrolizumab and placebo arms. The parameter values were based on the lower and upper limits used in one-way sensitivity analysis (eTable 2) and were further divided into every 25-percentile whenever appropriate. The values inside each colored block represent the ICER (US$ per quality-adjusted life-year, QALY) for pembrolizumab vs placebo. Bold-italic, larger values represent the combination(s) that the pembrolizumab vs placebo is cost-effective under the willingness-to-pay threshold at US$150,000/QALY.

eFigure 2A. Two-way sensitivity analysis on ICER by median overall survival (OS) of pembrolizumab and Placebo arms

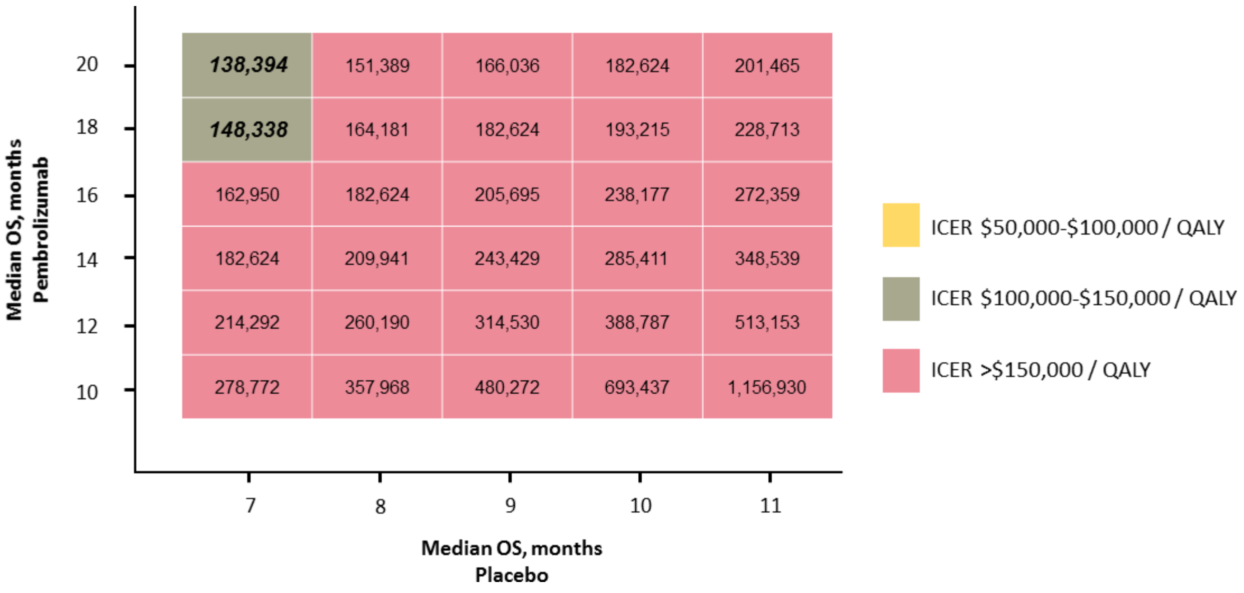

© 2021 Chiang, C-l, et al. *JAMA Network Open.*

eFigure 2B. Two-way sensitivity analysis on ICER by utility value of pembrolizumab and Placebo

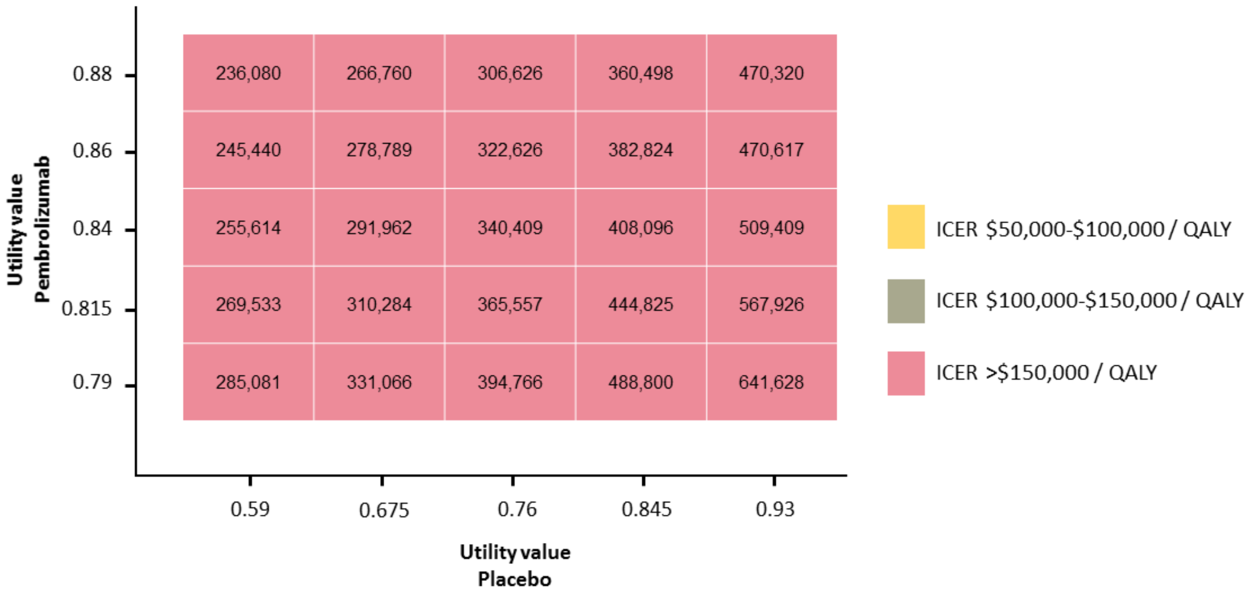

© 2021 Chiang, C-l, et al. *JAMA Network Open.*

eFigure 2C. Two-way sensitivity analysis on ICER by post-progression therapy costs of pembrolizumab and placebo arms

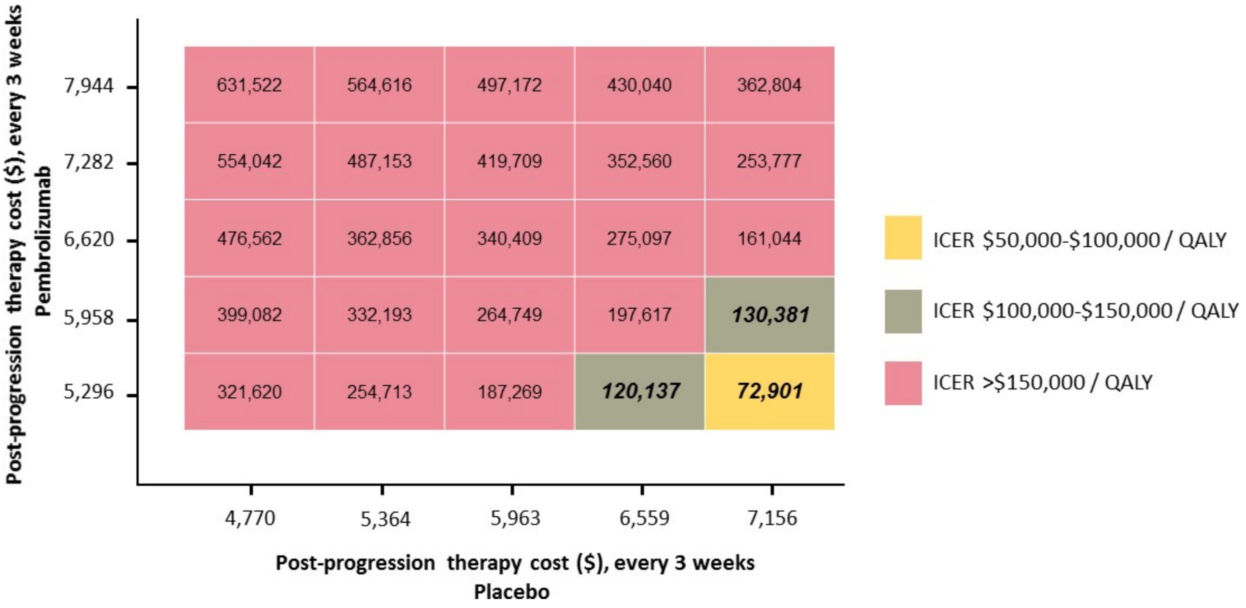

© 2021 Chiang, C-l, et al. *JAMA Network Open.*

eTable 4. Results of subgroup analyses

					Results		
	Sample size		Reported HRs			Cost-effectiveness acceptability at WTP	
**Subgroups**	**Pembro *N* = 278**	**Placebo *N* = 135**	**HR for PFS (95% Cl)**	**HR for OS (95% Cl)**	**ICER ($/QALY) (95% Cl)** [a]	**$100,000/QALY**	**$150,000/QALY**
**Sex**							
Male	226	112	0.74 (0.57 to 0.95)	0.76 (0.58 to 1)	332,627 (207,809 to 1,695,494)	0%	0%
Female	52	23	0.59 (0.33 to 1.06)	0.8 (0.44 to 1.47)	310856 (167,735 to –417,573)	0%	0%
**Geographic location**							
Asia excluding Japan	67	31	0.53 (0.33 to 0.86)	0.59 (0.37 to 0.95)	204,620 (158,947 to 829,781)	0%	0%
Non-Asia plus Japan	211	104	0.78 (0.6 to 1.02)	0.86 (0.64 to 1.14)	474,205 (230,117 to –1,916,945)	0%	0%
**ECOG**							
0	162	71	0.69 (0.5 to 0.94)	0.73 (0.52 to 1.03)	295,221 (183,040 to 2,940,530)	0%	0%
1	116	64	0.82 (0.57 to 1.17)	0.95 (0.66 to 1.37)	434,304 (234,364 to –455,554)	0%	0%
**MVI**							
Yes	36	16	0.80 (0.42 to 1.51)	0.57 (0.29 to 1.13)	213,200 (139,862 to –698,793)	0%	5.7%

© 2021 Chiang, C-l, et al. *JAMA Network Open.*

No	242	119	0.71 (0.55 to 0.90)	0.82 (0.63 to 1.06)	379,860 (220,133 to 2,937,393)	0%	0%
**AFP (ng/ml)**							
<200	129	58	0.64 (0.47 to 0.88)	0.68 (0.49 to 0.96)	254,765 (199,611 to 931,528)	0%	0%
≥200	149	77	0.82 (0.58 to 1.14)	0.88 (0.62 to 1.26)	544,509 (219,752 to –648,890)	0%	0%
**Sorafenib discontinuation reason**							
PD	242	117	0.73 (0.57 to 0.93)	0.73 (0.56 to 0.94)	304,824 (197,634 to 929,153)	0%	0%
Intolerance	36	18	0.55 (0.28 to 1.09)	1.26 (0.58 to 2.76)	882,786 (182,676 to –171,877)	0%	0%
**Extra-hepatic disease**							
Yes	195	93	0.67 (0.51 to 0.88)	0.86 (0.64 to 1.16)	400,816 (218,573 to –5,279,230)	0%	0%
No	83	42	0.91 (0.58 to 1.42)	0.67 (0.42 to 1.08)	286,814 (157,334 to –1,058,009)	0%	0%
**Viral status**							
HBV	72	29	0.7 (0.44 to 1.13)	0.57 (0.51 to 1.68)	208,381 (179,122 to –882,195)	0%	0%
HCV	43	21	0.46 (0.24 to 0.90)	0.96 (0.48 to 1.92)	326,144 (173,801 to –11,700)	0%	0%

© 2021 Chiang, C-l, et al. *JAMA Network Open.*

Uninfected	163	85	0.75 (0.56 to 1.01)	0.88 (0.64 to 1.2)	485,021 (225,073 to –1,181,058)	0%	0%
**BCLC stage**							
B	56	29	0.91 (0.53 to 1.57)	0.93 (0.51 to 1.68)	830,058 (181,150 to –251,108)	0%	0%
C	222	106	0.67 (0.52 to 0.86)	0.77 (0.58 to 1.01)	318,829 (200,754 to 1,257,013)	0%	0%

Abbreviations: AFP, alpha-fetoprotein; BCLC, Barcelona Clinic Liver Cancer; CI, confidence interval; ECOG, Eastern Cooperative Group; HBV, hepatitis B virus; HCV, hepatitis C virus; HR, hazard ratio for pembrolizumab vs placebo; ICER, incremental cost-effectiveness ratio; PD, disease progression; Pembro, pembrolizumab; MVI, macrovascular invasion; OS, overall survival; PFS, progression-free survival; QALY, quality-adjusted life-year; WTP, willingness-to-pay

[a] Negative ICERs refer to worse health benefit (i.e., negative incremental QALY) for pembrolizumab vs placebo

© 2021 Chiang, C-l, et al. *JAMA Network Open.*
